# *C. elegans mpk-1b* long first intron enhances MPK-1B protein expression

**DOI:** 10.17912/micropub.biology.000350

**Published:** 2021-01-14

**Authors:** Sarah Robinson-Thiewes, Judith Kimble

**Affiliations:** 1 University of Wisconsin-Madison: Department of Genetics, Madison, WI USA; 2 University of Wisconsin-Madison: Department of Biochemistry, Madison, WI USA; 3 University of Wisconsin-Madison: Department of Medical Genetics, Madison, WI USA

**Figure 1. The mpk-1b long first intron enhances MPK-1B translation:**
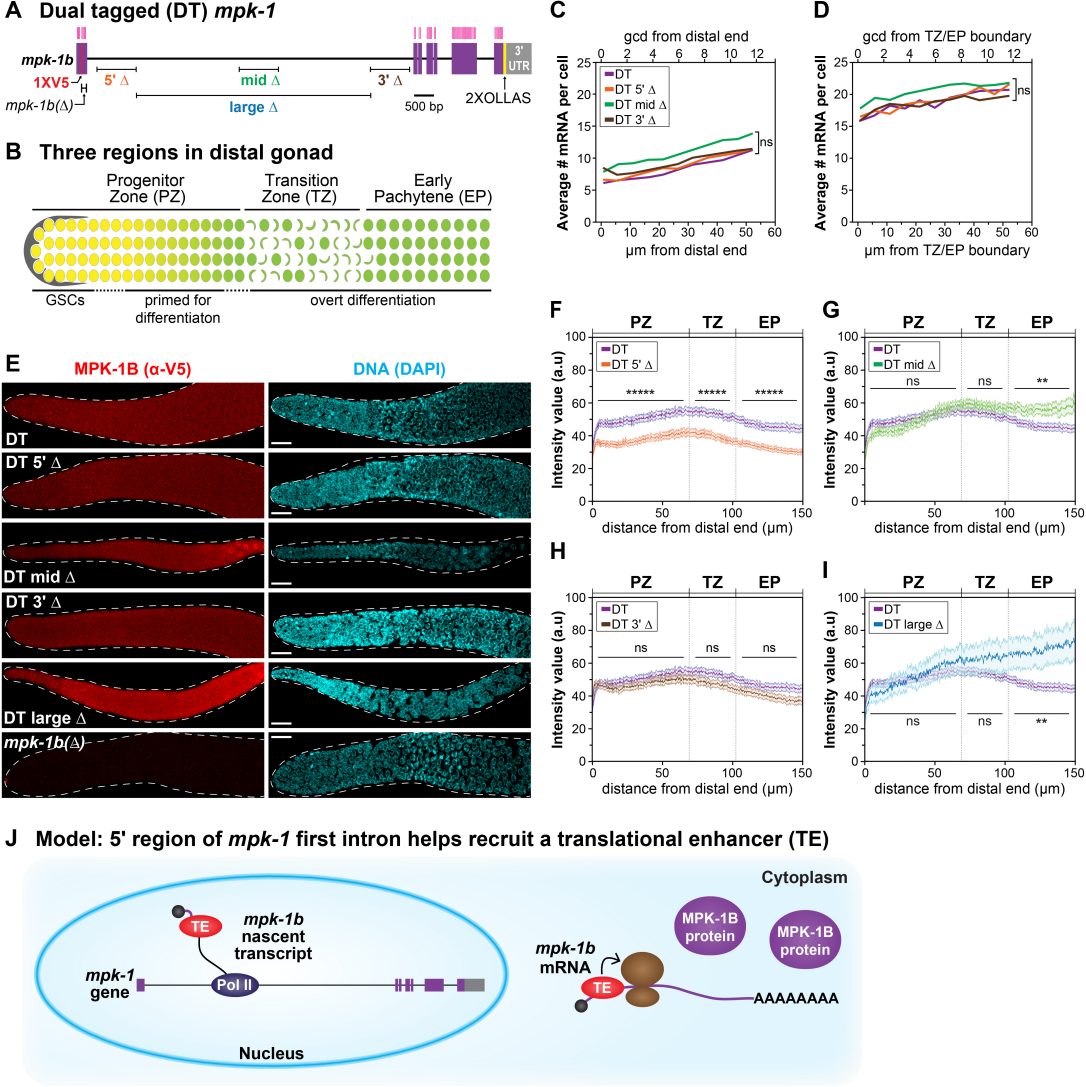
**(A)** The *mpk-1* locus generates two RNAs, *mpk-1b* and *mpk-1a;* for simplicity, only *mpk-1b* is shown with its exclusive first exon and ~8 kb long first intron. Deletions within the long first intron were generated in the dual tagged (DT) *mpk-1* locus*,* which carries 1XV5 to mark MPK-1B specifically, and 2XOLLAS to mark both MPK-1A and MPK-1B (Robinson-Thiewes *et al.* 2020a). A small deletion within the first exon, *mpk-1b(Δ)*, abolishes production of MPK-1B (Robinson-Thiewes *et al.* 2020a). One smFISH probe set targets *mpk-1b* exons (magenta vertical lines) and a second (not shown) marks the long first intron, as previously described (Robinson-Thiewes *et al.* 2020b). Purple boxes, exons; gray boxes, UTRs. **(B)** Three regions in the distal gonad: Progenitor Zone (PZ), Transition Zone (TZ), and Early Pachytene region (EP). Niche, grey; proliferative germ cells yellow; meiotic germ cells green. **(C-D)** Average number of mRNA per cell as a function of distance in PZ (C) and EP (D). x-axes, germ cell diameters (gcd)(top) or µm (bottom) from either the distal end of the PZ (C) or from the TZ/EP boundary; y-axes. DT *mpk-1* intron mutants were compared to DT *mpk-1* using the student’s t-test. ns, not significant. Number gonads scored in PZ: DT, 28; DT 5’Δ, 39; DT mid Δ, 29; DT 3’Δ, 39; and in EP: DT, 27; DT 5’Δ, 37; DT mid Δ, 29; DT 3’Δ, 39. See methods for additional details. **(E)** Representative images of stained dissected gonads, with MPK-1B detected using α-V5 and nuclei detected with DAPI. Maximum projection shown. Scale bar, 15 µm. **(F-I)** MPK-1B protein quantification as a function of distance across the PZ, TZ and EP. x-axis: top, germline region; bottom, µm from distal end; y-axis, fluorescence intensity normalized to that in *mpk-1b(Δ),* which makes no MPK-1B. Each DT intron mutant is displayed together with the DT *mpk-1* control; student’s t-test was used to determine statistical significance. **, p<0.0001; *****, p<0.0000001; ns, not significant. Number gonads scored: DT, 47; DT 5’Δ, 37; DT mid Δ, 26; DT 3’Δ, 36; DT large Δ, 16. See methods for details. **(J)** Model that 5’ region of the *mpk-1b* long first intron enhances MPK-1B translation by recruiting a translational enhancer. RNA polymerase II, Pol II; ribosome, brown circles. Not to scale.

## Description

Most eukaryotic genes make nascent transcripts that include both introns and exons. Introns are spliced out, usually co-transcriptionally, to generate mature mRNA (Herzel *et al.* 2017). Once considered a byproduct of transcription, introns are now known to affect virtually all aspects of mRNA metabolism, from transcriptional initiation to translation (Swinburne and Silver 2008; Chorev and Carmel 2012; Rose 2019). Genes can have multiple introns of variable size, but the first intron is often the longest (Smith 1988; Kriventseva and Gelfand 1999; Bradnam and Korf 2008). We wondered if the long first intron of a critical developmental regulator might affect its expression during development. To address this question, we analyzed expression of the *C. elegans mpk-1b* RNA, which encodes a nematode ERK/MAPK ortholog and has a long first intron (Figure 1A). Because *mpk-1b* is a germline-specific RNA (Lee *et al.* 2007; Robinson-Thiewes *et al.* 2020a), we assayed its expression along the developmental axis of the distal gonad (Figure 1B). Here, germ cells move through three regions as they progress from germline stem cells (GSCs) to differentiation: GSCs and GSC daughters primed for differentiation reside in the Progenitor Zone (PZ); germ cells enter meiotic prophase in the Transition Zone (TZ); and continue through meiotic prophase in the Early Pachytene (EP) region (Figure 1B)(Hubbard and Schedl 2019).Previously, we reported that a set of three ~1 kb deletions at distinct sites within the *mpk-1b* longintron did not affect its transcription or mRNA abundance (Robinson-Thiewes *et al.* 2020b). Here, we report effects of these deletions on production of the MPK-1B protein.

Previously we characterized deletions within the long first intron in an untagged *mpk-*1locus (Robinson-Thiewes *et al.* 2020b), and in a separate work, we generated an *mpk-1* locus with two epitope tags—dual tagged (DT) *mpk-1* (Robinson-Thiewes *et al.* 2020a). To assay effects of the intron deletions on MPK-1B protein production, we generated them in DT *mpk-1*. The three smaller deletions are each ~1 kb in length, while a larger deletion is 5.7 kb (Figure 1A). DT 5’Δ removes an *in silico-*predicted hair pin loop; DT mid Δ removes an enhancer for the somatic *mpk-1a* transcript; and DT 3’Δ removes a region with no distinguishing features, as described previously (Robinson-Thiewes *et al.* 2020b).

First, we asked if the DT deletions affect *mpk-1b* mRNA abundance. We performed single molecule in situ hybridization (smFISH) in gonads homozygous for each deletion, followed by MATLAB analysis of mRNA abundance as a function of distance along the developmental axis (see Methods) (Robinson-Thiewes *et al.* 2020b); the MATLAB code did not allow analysis of DT large Δ (see Methods). For each variant, we scored mRNA abundance in the distal PZ (12 rows from distal end) (Figure 1C) and in the EP (12 rows from TZ/EP boundary) (Figure 1D) and compared results for each DT deletion with the DT *mpk-1* control. The mRNA abundance per cell did not differ statistically between any of the intron deletions and the control, as expected from analyses of the same deletions in the untagged *mpk-1* locus (Robinson-Thiewes *et al.* 2020b). We conclude that mRNA abundance is not affected by the various intron deletions.

Second, we asked if the DT intron deletions affect MPK-1B protein abundance. To this end, we used V5 antibody to detect MPK-1B in the distal gonad (Figure 1B) of DT *mpk-1,* DT *mpk-1b(Δ)*,and all four DT intron mutants (Figure 1E). Briefly, we quantified MPK-1B fluorescence using ImageJ (see Methods) and normalized intensities to DT *mpk-1b(Δ),* which does not make MPK-1B (Figure 1E-I)*.* MPK-1B abundance was lower in DT 5’Δ than DT MPK-1B in all three germline regions (Figure 1F), whereas MPK-1B did not differ in DT 3’Δ and the control (Figure 1H). In DT mid Δ and DT large Δ, the MPK-1B abundance was not statistically different from control in the PZ and TZ, but was higher in the EP (Figure 1F, 1J). Because both mid Δ and large Δ remove an enhancer of somatic *mpk-1a* (Robinson-Thiewes *et al.* 2020b), we favor the idea that this EP effect is due to altered somatic MPK-1A activity, and hence is an indirect result of defective feedback on the germline; alternatively, the effect could be direct with the mid Δ and large Δ lowering MPK-1B translation autonomously in the EP region. Additional experiments are required to distinguish between these two possibilities. We conclude that MPK-1B protein abundance is decreased in 5’Δ gonads compared to control. Because mRNA abundance is unaffected in 5’Δ gonads (Figures 1C-D), the simplest conclusion is that the 5’Δ deletion changes translation of the *mpk-1b* mRNA.

We do not understand how the *mpk-1b* long first intron enhances translation of the *mpk-1b* mRNA but note that other examples occur throughout Eukarya (Shaul 2017). The mechanism is also not fully understood for other examples, but one model is that a translational enhancer, perhaps the exon junction complex (EJC), joins the nascent transcript in the nucleus and moves with the mature mRNA to the cytoplasm where it enhances translation (Chazal *et al.* 2013; Heath *et al.* 2016; Hir *et al.* 2016). By analogy, we speculate that a translational enhancer associates with the *mpk-1b* nascent transcript in the nucleus and moves with *mpk-1b* mRNA to the cytoplasm where it promotes translation (Figure 1J).

## Methods

Strains and growth conditions: All strains were maintained at 20°C on OP50 seeded plates. All strains will be available at the CGC.

**Table:** 

Strain number	Genotype	Shorthand
N2	wildtype	WT (untagged)
JK6383	*mpk-1(q1183) III*	DT
JK6406	*mpk-1(q1204) III*	DT 5’Δ
JK6404	*mpk-1(q1202) III*	DT mid Δ
JK6407	*mpk-1(q1208) III*	DT 3’Δ
JK6452	*mpk-1(q1206) III*	DT large Δ
JK6403	*mpk-1(q1201) III/qC1[qIs26] III*	*mpk-1b(Δ)/*Balancer

**CRISPR-induced intron deletions in *mpk-1*DT:** All deletionswere made using published crRNAs and protocols (Robinson-Thiewes *et al.* 2020b).

**smFISH, imaging, and MATLAB analysis:** Young adult hermaphrodites (L4+12 hr) were dissected and stained with *mpk-1* smFISH using both intron and exon probe sets, as previously described (Robinson-Thiewes *et al.* 2020a). Because the MATLAB code requires signals from both intron and exon probes, mRNA abundance could not be assessed for DT large Δ, which removes most intron probe binding sites. Individual smFISH exon and intron probe set sequences, smFISH control images, and untagged intron deletion data are published (Robinson-Thiewes *et al.* 2020b). After staining, gonads were mounted in Prolong Glass, sealed with VALAP, and stored at -20°C. We used a Leica SP8 confocal microscope and the same imaging conditions previously described (Robinson-Thiewes *et al.* 2020b). Images were analyzed in MATLAB using the same procedure and code as originally described (Robinson-Thiewes *et al.* 2020b).

**MPK-1B staining, imaging, and quantification:** Young adult hermaphrodites (L4+12 hr) were dissected and stained as described (Robinson-Thiewes *et al.* 2020a). Mouse α-V5 (Bio-Rad), diluted 1:1000, was used for the primary antibody; α-mouse alexa555, diluted 1:1000, was used for the secondary antibody. DAPI (1 µg/mL) was diluted 1:1000. Gonads were mounted in Prolong Glass antifade, sealed with VALAP, and stored at -20°C. Gonads were imaged on a Leica SP8 confocal microscope following published procedure (Robinson-Thiewes *et al.* 2020a). Images were imported into ImageJ and the fluorescence quantification followed published protocol (Robinson-Thiewes *et al.* 2020a). ImageJ data was exported into MATLAB for downstream analyses.

**Replicates:** Two replicates were performed for each genotype and each experiment reported. Replicates were raised in parallel on separate plates. For each experiment, replicates were dissected, stained, and imaged in parallel. Because data from the replicates were not statistically significant from each other, they were combined for presentation.

· smFISH in PZ: DT replicate 1, 14; DT replicate 2, 14; DT 5’Δ replicate 1, 20; DT 5’Δ replicate 2, 19; DT mid Δ replicate 1, 15; DT mid Δ replicate 2, 14; DT 3’Δ replicate 1, 19; DT 3’Δ replicate 2, 20.

· smFISH in EP: DT replicate 1, 13; DT replicate 2, 14; DT 5’Δ replicate 1, 18; DT 5’Δ replicate 2, 19; DT mid Δ replicate 1, 15; DT mid Δ replicate 2, 14; DT 3’Δ replicate 1, 19; DT 3’Δ replicate 2, 20.

· Immunostaining: DT replicate 1, 23; DT replicate 2, 24; DT 5’Δ replicate 1, 19; DT 5’Δ replicate 8, 19; DT mid Δ replicate 1, 20; DT mid Δ replicate 2, 6; DT 3’Δ replicate 1, 18; DT 3’Δ replicate 2, 18; DT large Δ replicate 1, 9; DT large Δ replicate 2, 7.

**MATLAB analyses:** smFISH images were analyzed using codes available at https://github.com/robinson-thiewes/Robinson-thiewes-PNAS-codes- (DOI: 10.5281/zenodo.4007918). All statistical tests used in this work were performed in MATLAB using the student’s t-test ttest2 function. Shaded bar graphs in Figure 1F-I were generated using shadedErrorBar (https://github.com/raacampbell/shadedErrorBar).
